# Comprehensive analysis of microRNAs in breast cancer

**DOI:** 10.1186/1471-2164-13-S7-S18

**Published:** 2012-12-07

**Authors:** Hong-Tai Chang, Sung-Chou Li, Meng-Ru Ho, Hung-Wei Pan, Luo-Ping Ger, Ling-Yueh Hu, Shou-Yu Yu, Wen-Hsiung Li, Kuo-Wang Tsai

**Affiliations:** 1 Department of Surgery, Kaohsiung Veterans General Hospital, Kaohsiung, Taiwan, Republic of China; 2 Department of Emergency, Kaohsiung Veterans General Hospital, Kaohsiung, Taiwan, Republic of China; 3 Genomics Research Center, Academia Sinica, Taipei, Taiwan, Republic of China; 4 Biodiversity Research Center, Academia Sinica, Taipei, Taiwan, Republic of China; 5 Department of Medical Education and Research, Kaohsiung Veterans General Hospital, Kaohsiung, Taiwan, Republic of China; 6 Institute of Biomedical Sciences, Academia Sinica, Taipei, Taiwan, Republic of China; 7 Department of Ecology and Evolution, University of Chicago, Chicago, IL 60637, USA; 8 Department of Biotechnology, Tajen University, Taiwan, Republic of China

## Abstract

**Background:**

MicroRNAs (miRNAs) are short noncoding RNAs (approximately 22 nucleotides in length) that play important roles in breast cancer progression by downregulating gene expression. The detailed mechanisms and biological functions of miRNA molecules in breast carcinogenesis have yet to be fully elucidated. This study used bioinformatics and experimental approaches to conduct detailed analysis of the dysregulated miRNAs, arm selection preferences, 3' end modifications, and position shifts in isoforms of miRNAs (isomiRs) in breast cancer.

**Methods:**

Next-generation sequencing (NGS) data on breast cancer was obtained from the NCBI Sequence Read Archive (SRA). The miRNA expression profiles and isomiRs in normal breast and breast tumor tissues were determined by mapping the clean reads back to human miRNAs. Differences in miRNA expression and pre-miRNA 5p/3p arm usage between normal and breast tumor tissues were further investigated using stem-loop reverse transcription and real-time polymerase chain reaction.

**Results:**

The analysis identified and confirmed the aberrant expression of 22 miRNAs in breast cancer. Results from pathway enrichment analysis further indicated that the aberrantly expressed miRNAs play important roles in breast carcinogenesis by regulating the mitogen-activated protein kinase (MAPK) signaling pathway. Data also indicated that the position shifts in isomiRs and 3' end modifications were consistent in breast tumor and adjacent normal tissues, and that 5p/3p arm usage of some miRNAs displayed significant preferences in breast cancer.

**Conclusions:**

Expression pattern and arm selection of miRNAs are significantly varied in breast cancers through analyzing NGS data and experimental approach. These miRNA candidates have high potential to play critical roles in the progression of breast cancer and could potentially provide as targets for future therapy.

## Background

Breast cancer is one of the major causes of cancer-related deaths worldwide and the most common cancer among women [1]. Metastatis to distant organs and lymph nodes represents a major problem, usually leading to high mortality. The investigation of breast cancer-associated genes for early detection or therapeutic targeting could potentially improve the survival rates of breast cancer patients.

MicroRNAs (miRNAs) are small RNA molecules with important regulatory functions in several physiological activities [2]. MicroRNAs are processed from primary transcripts (pri-miRNAs) in 2 maturation steps. First, the pri-miRNAs are processed by Drosha, forming the precursor miRNAs (pre-miRNAs), composed of a 5p arm, a 3p arm, and a terminal loop, approximately 70 nucleotides in length. Following the transport of pre-miRNAs to the cytoplasm by exportin 5, they are further processed by Dicer to release the terminal loop and the duplex (5p arm/3p arm), 22 nucleotides in length. The 5p arm/3p arm of the duplex is unwound at the end because of weaker hydrogen binding. The 5p or the 3p arm is selectively loaded into the RNA-induced silencing complex (RISC) and serves as mature miRNA [3-5]. Recent studies described a phenomenon in which RNA editing or nucleotide addition generated 3' end sequence variants of miRNAs [6-13]. Fernandez-Valverde et al. [6] reported that miR-282 and miR-312a are enriched for 3' adenosine additions during early embryonic development, which increases miRNA stability or enhances miRNA and mRNA interaction.

MicroRNAs exert their effects by repressing their target genes. They downregulate target gene expression by repressing translation or by degrading mRNAs. Previous studies reported that miRNAs play important roles in the oncogenesis pathway [14-18]. The tumor-associated miRNAs were either tumor repressors, preferentially expressed in normal tissue, or onco-miRNAs, preferentially expressed in tumor tissue. These are aberrantly expressed in human breast cancer, including miR-9, miR-21, miR-31, miR-34a, miR-155, miR-200, miR-205, miR-206, and miR-335 [19-22]. Although several studies have investigated the functions of miRNAs in breast tumors, these only included a small fraction of existing miRNAs [23-26]. Using miRNA profiling approach, numerous breast cancer-associated miRNAs were identified [19,27-30]. Ryu et al. [27] identified 189 candidate novel microRNAs in human breast cancer cell lines by deep sequencing technology. Therefore, emerging NGS technologies can be used not only to identify novel miRNAs, but can also be applied in several miRNA-associated studies.

In the studies using NGS data for miRNA profiling, it is usually observed that miRNA sequence reads exist as isoforms, named isomiRs, with position and length shift compared with the reference miRNAs [31]. Recently, more and more studies worked on the isomiR issues, such as isomiR pattern preferences in specific libraries, target gene selection difference between different isomiRs and so on [6,32]. Therefore, NGS data provides a good resource for miRNA expression profiling and isomiR related studies. In 2011, Farazi et al., [33] used NGS data to determine miRNA expression profiles in breast tissues with differing tumor malignancies. They focused on the relevance of specific miRNAs and the tumor malignancy type, without providing further experimental validation. The present study applied their NGS data to conduct analysis of miRNA-associated changes in breast cancer, including differential miRNA expression, position shifts in isomiRs, 3' end modifications, and arm selection preferences of pre-miRNAs.

## Materials and methods

### Collection and preprocessing of sequence reads

The small RNA transcriptome data of breast tumor (accession number: SRP006574) was downloaded from the NCBI Sequence Read Archive (SRA). These data included more than 200 samples and was classified into 2 libraries: normal and tumor (invasive ductal carcinoma). The initial sequence reads were subjected to 3' adaptor trimming to generate the clean reads, as described previously [10,13]. For higher confidence, only the clean reads with read count ≥ 2 were included in further analysis.

### Mapping clean reads to pre-miRNAs

MicroRNA expression profiles in different libraries were determined by mapping the clean reads back to human pre-miRNAs (miRBase 17). Several miRNA genes show high similarity (such as 68 mir-548 paralogous miRNAs in humans). This results in multiple ambiguous hits when mapping a read back to human miRNAs if variations are allowed. To eliminate the ambiguous mapping hits, no mismatch was allowed during the mapping procedure. Previous reports described observing nucleotide additions at the 3' ends of miRNAs [31,34-36] that could cause mismatches at the terminal of the mapping alignment. In order to follow the no-mismatch policy and keep the 3' end variation, like the method in Fernandez-Valverde's study [6], we trimmed out the terminal 3' end mismatch one by one until the perfect match reads were at least 18 nucleotides in length. By doing so, we can keep not only an at least 18-nt perfect alignment but also the 3' end variations.

### Classifying non-miRNA reads into different data sets

The non-miRNA sequence reads were further classified into 9 classes by mapping to different data sets with bowtie [37], allowing a single nucleotide variation. The sequences of mRNAs and other ncRNAs were derived from NCBI RefSeq 47 [38]. The sequences of tRNAs was downloaded from the Genomic tRNA database [39]; sequences of rRNAs were downloaded from the SILVA database [40]. The sequences of snoRNAs, scaRNAs, and snRNAs were all downloaded from NONCODE [41]. The sequence reads not belonging to any of the described RNA classes were uploaded to RepeatMasker for identification of repeat elements and classified as unknown.

### Samples and RNA extraction

Ten paired (tumor and adjacent normal) samples were collected from breast cancer patients receiving surgical operation at the Department of Surgery, Kaohsiung Veterans General Hospital. The total RNA of tissue was extracted using a TRIzol reagent (Invitrogen, USA), according to the instruction manual. Briefly, tissue samples were homogenized in 1 ml of TRIzol reagent and mixed with 0.2 ml chloroform to extract protein; RNA was precipitated using 0.5 ml isopropanol. The concentration, purity, and amount of total RNA were determined using a Nanodrop 1000 spectrophotometer (Nanodrop Technologies Inc., USA).

### Stem-loop reverse transcription (RT) and real-time PCR

Reverse transcription primers were specifically designed for the examined miRNAs according to the methods reported by Chen et al., [42]. One microgram of total RNA was reverse transcribed in a stem-loop RT reaction with RT primers and SuperScript III Reverse Transcriptase according to the user's manual (Invitrogen, Carlsbad, CA, USA). The reaction was performed under the following incubation conditions: 30 min at 16°C, followed by 50 cycles of 20°C for 30 s, 42°C for 30 s, and 50°C for 1 s. The enzyme was subsequently inactivated by incubating at 85°C for 5 min. Real-time PCR reactions were performed using an miRNA-specific forward primer and a universal reverse primer with incubation at 94°C for 10 min, followed by 40 cycles of 94°C for 15s and 60°C for 32 s. Gene expression levels were detected using SYBR Green I assay (Applied Biosystems, Foster City, CA, USA), and miRNA expression levels were normalized to that of U6. The primer sequences for the examined miRNAs are listed in Additional File 1.

### Pathway enrichment analysis

Human miRNA target gene data was downloaded from TargetScan 6.0. The target genes of differentially expressed miRNAs were extracted, then mapped onto KEGG pathways based on the enzyme commission (EC) numbers using the R package SubPathwayMiner v.3.1 [43]. The hypergeometric test was then performed to identify significantly enriched pathways and calculate the false positive discovery rate in FDR-corrected q-value.

## Results and discussion

### Analysis of miRNA sequence reads

After subjecting the downloaded small RNA reads to the 3' adaptor trimming procedure, the normal library contained approximately 2.7 million clean reads and the tumor library approximately 38.3 million clean reads (Table 1). Using the mapping criteria, the clean reads were mapped back to human pre-miRNAs (miRBase 17). Approximately 75% of the normal clean reads and 85% of the tumor clean reads belonged to miRNAs. The detection of a greater number of miRNA and pre-miRNAs in the tumor library than in the normal library reflected greater sequencing depth in the tumor library. The libraries contained an unequal number of initially used reads; therefore, comparisons in miRNA expression between the libraries were performed using the unit of transcript per million (TPM). There were no significant differences in the expression levels of most miRNAs between normal and tumor tissues (Additional File 2).

**Table 1:** Categories of sequence reads in the 2 breast libraries.

Library	Normal	Tumor
# clean reads	2 785 848	38 335 412
% miRNA reads	75.24%	84.79%
# detected pre-miRNAs	455	689
# detected miRNAs	631	906
# detected miRNAs at opposite arm	54	150
% miRNA reads with 3' end modification	17.65%	12.45%

According to miRBase 17 annotation, there are 1424 human pre-miRNAs, encoding 1733 mature miRNAs. These pre-miRNAs and mature miRNAs result in 1902 pairs of pre-miRNA/miRNA.

The clean reads not defined as miRNAs were further classified into an additional 9 classes by mapping to other transcript sequences. As shown in Table 2, in most categories, the clean read distributions in the 2 libraries were similar. However, compared with the normal library, the tumor library had a higher percentage of miRNA reads but lower percentages of other ncRNA and unknown reads. After further investigation, more than 85% of the unknown category (data not shown) in the 2 libraries could not be mapped back to the human genome (hg19). Our previous study showed that such unmappable reads could be derived from the exon-exon junctions of novel alternative splicing transcripts or from the transcripts of infection viruses [44]. These unmappable reads warrant further investigation.

**Table 2:** Categories of clean sequence reads in the 2 breast libraries

Category	miRNA	mRNA	tRNA	rRNA	snoRNA	scaRNA	snRNA	other ncRNA	repeat	unknown
Normal	75.24%	2.16%	0.47%	0.87%	0.84%	0.04%	0.12%	9.78%	0.0349%	10.45%
Tumor	84.79%	0.86%	0.37%	0.33%	0.35%	0.04%	0.14%	6.40%	0.0006%	6.70%

### Differentially expressed miRNAs

**Figure 1:**
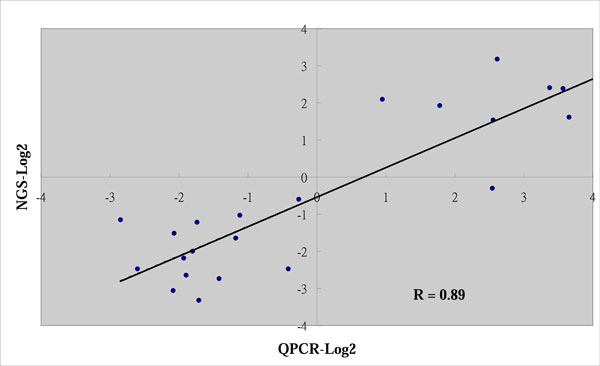
**Comparison of fold changes in expression determined using NGS analysis and PCR**. The scatter plot shows that NGS and PCR results were highly correlated, with a Pearson correlation coefficient of 0.89

A comparison of the miRNA expression levels in normal and tumor breast tissues provided a list of differentially expressed miRNAs. Similar to other high throughput technologies, NGS data is susceptible to background noise, producing biased results. Stem-loop RT-PCR was thus used to further validate the 23 miRNAs with the highest fold changes in expression levels between normal breast and breast tumor tissues. Stem-loop RT-PCR reportedly shows a high correlation with NGS technologies [10,45]. In common with RT-PCR assays, an internal control is used for comparing the same genes in different samples. In NGS assays, no such internal control is used. The fold changes in the same miRNAs between normal and tumor breast tissues, as detected using PCR and NGS, were thus compared. Among the 23 evaluated miRNAs, the fold changes detected using PCR and NGS were highly correlated, with a Pearson correlation coefficient of 0.89 (Figure 1). This was a similar finding to those of previous studies.

Among the 23 examined miRNAs, 15 are preferentially expressed in normal tissue, whereas 8 are preferentially expressed in tumor tissue (Table 3). The tissue preferences of miRNAs determined using NGS are consistent with those identified using PCR with only one exception: hsa-miR-423-3p. The present study, therefore, generated an authentic list of differentially expressed miRNAs in breast cancer using an experimental approach.

**Table 3:** Validation of differentially expressed miRNAs using PCR.

miRNA	NGS normal	NGS tumor	NGS fc	PCR fc	Consistent?
has-let-7b	32 594.1	16 107.1	0.49	0.46	Y
hsa-let-7c	7 710.1	2 439.1	0.32	0.44	Y
hsa-miR-22	69 532.1	24 576.1	0.35	0.24	Y
hsa-miR-125b	44 624.1	6 613.1	0.15	0.37	Y
hsa-miR-143	38 419.1	17 359.1	0.45	0.14	Y
hsa-miR-144	3 548.1	351.1	0.10	0.30	Y
hsa-miR-145	28 682.1	6 435.1	0.22	0.26	Y
hsa-miR-193a-5p	1 836.1	298.1	0.16	0.27	Y
hsa-miR-193b	2 469.1	1 058.1	0.43	0.30	Y
hsa-miR-199b-5p	1 608.1	1 055.1	0.66	0.83	Y
hsa-miR-320a	16 734.1	3 060.1	0.18	0.75	Y
hsa-miR-378	12 610.1	1 542.1	0.12	0.24	Y
hsa-miR-497	8 005.1	2 010.1	0.25	0.29	Y
hsa-miR-99a	31 360.1	5 548.1	0.18	0.16	Y
hsa-miR-141	5007.1	14 541.1	2.90	5.88	Y
hsa-miR-375	240.1	2 372.1	9.88	37.95	Y
hsa-miR-425	592.1	2 529.1	4.27	1.93	Y
hsa-miR-203	197.1	1 045.1	5.30	9.80	Y
hsa-miR-342-3p	350.1	1 331.1	3.80	3.43	Y
hsa-miR-182	316.1	1 653.1	5.23	11.86	Y
hsa-miR-200b	1 362.1	4 162.1	3.06	12.61	Y
hsa-miR-183	118.1	1 067.1	9.04	6.12	Y
hsa-miR-423-3p	2 446.1	1 993.1	0.81	5.96	N

NGS normal and NGS tumor denote miRNA relative read counts (TPM) in normal and tumor tissues. The NGS fold change (NGS fc) values are the quotients of miRNA read counts in normal tissue divided by those in tumor tissue. The PCR fold change (PCR fc) values are the quotients of miRNA expression levels, relative to U6 in normal tissue, divided by those in tumor tissue, averaged from 4 independent experiments

### Enrichment analysis of miRNA-involved pathway

Following the identification of the differentially expressed miRNAs, the subsequent stage was to identify their functions as defined by their target genes. Several computational methods can be used to identify the putative target genes of miRNAs [46-49]. However, these computational methods typically depend on the hydrodynamic stability of the miRNA/3'UTR duplex, and usually produce several false positive results. The most recently developed method is dependent on computational identification and also on the enrichment analysis of a target gene pathway [50]. The present study applied the same strategy and did the analysis on the miRNAs with the following features: with high expression alteration and high expression level in at least one library. So, we selected the union of the target genes of hsa-miR-141 and hsa-miR-200b for tumor-preferring miRNAs, and the union of the target genes of hsa-miR-22, hsa-miR-125b, and hsa-miR-99a for normal-preferring miRNAs. The 2 unions of genes were individually subjected to pathway enrichment analysis. The pathway enrichment analysis result showed that the target genes of tumor-preferring miRNAs are significantly enriched in the mitogen-activated protein kinase (MAPK) pathway, with a *p*-value of 2.1E-6 (Additional file 3); while, the target genes of normal-preferring miRNAs are significantly enriched also in the mitogen-activated protein kinase (MAPK) pathway, with a *p*-value of 2.4E-6 (Additional file 4).

The MAPK pathways are highly conserved kinase modules involved in fundamental cellular processes such as growth, proliferation, migration, and apoptosis [51]. Studies have identified that the miR-200 family (miR-200a, miR-200b, miR-200c, miR-141 and miR-429) is overexpressed in breast cancer, promoting breast cancer metastasis and drug resistance [25,52]. Studies have also reported that overexpression of miR-200 in epithelial cell lines leads to an inhibition of transforming growth factor-β (TGF-β) and induction of epithelial mesenchymal transition (EMT) [53,54]. These results indicated that the miR-200 family plays dual roles in modulating breast cancer metastasis through the regulation of the complex MAPK signaling pathway. However, miR-99a plays opposite roles in the regulation of cancer progression. Oneyama et al. [55] reported that overexpression of miR-99a led to the suppression c-Src-transformed cell growth, by controlling the mTOR/FGFR3 pathway in various human cancers. In epithelial NMUMG cells, however, miR-99a promoted proliferation and migration by regulating TGF-β-induced breast EMT [56]. Imbalance in the MAPK signaling pathway can, therefore, lead to promotion or inhibition of cancer cell progression. This data indicates that aberrant miRNA expression can result in the dysregulation of breast cancer cell proliferation, apoptosis, cell cycle, and migration through regulation of the component genes of the complex MAPK signaling pathway.

Previous studies usually focus on the regulation relationship between one miRNA versus one pathway. Since many miRNAs are simultaneous up- or down-regulation in the same tissue, we are curious if they simultaneously act on the same pathway. So, we pick up the target gene unions of up- or down-regulated miRNAs, followed by pathway enrichment analysis on the target genes of the same union. Our result showed that the simultaneously up-regulated or down-regulated miRNAs execute their functions by acting on the same MAPK pathway. Actually, we also did the pathway enrichment analysis as usual, one miRNA versus one pathway. And, we got the same conclusion that the simultaneously up- or down-regulated miRNAs simultaneously act on the same pathway.

### MicroRNA 3' end additional nontemplate nucleotides in breast cancer

**Figure 2:**
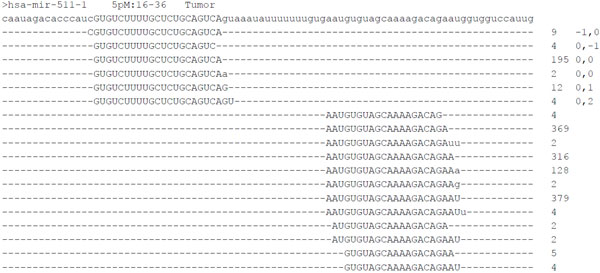
**Mapping results of hsa-miR-511-1**. As shown in 5p:16-36, hsa-miR-511-1 encodes mature miRNA at its 5p arm only and miRNA spans from nucleotide 16 to 36 of the hairpin. The integer values on the left denote the read count of each isomiR. The comma-separated values denote the position shift in the isomiR relative to the miRBase annotated positions (16 to 36). The nucleotides in lowercase denote the sequence fragments originating from the 3' modification event (Additional file 5)

MicroRNAs reportedly undergo RNA editing or nucleotide addition at the 3' end, causing a mismatch at the termini of the mapping alignments [31,34-36]. As shown in Figure 2, the isomiRs of miR-511 have adenine (A) or uracil (U) added at their 3' ends. Therefore, the 3' end additional nontemplate nucleotides widely occur during the miRNA maturation process. Further investigation of the 3' end nucleotide modification events using the alternative mapping procedure showed that more than 12% of miRNA reads undergo 3' end nucleotide modification in all libraries (Table 1). As shown in Additional File 5, A and U accounted for approximately 80% of modification events. In addition, AA, UU, AU, cytosine (C), and guanine (G) contributed at least 1% of 3' end nucleotide modification events. The 3' end nucleotide modification event, therefore, prefers the A or U nucleotide. The same nucleotide modification preferences were displayed by normal breast and breast tumor tissues.

### Position shifts in isomiRs in breast cancer

It has been widely observed that miRNA exists as isoforms, or isomiRs, generated by a position shift during the maturation process [31]. The present study detected all isomiRs by mapping the clean reads back to pre-miRNAs. For example, hsa-miR-511 has 5 isomiRs, each with differing length. The expression of an miRNA can be derived by summarizing the read counts of its isomiRs (Figure 2). Morin et al. [31] showed that the miRBase reference miRNA (the isomiR with position shift 0,0 in Figure 2) is not always the most abundant isomiR. Among the detected miRNAs in libraries, approximately 55% are the most abundant isomiRs; most of the remaining miRNAs are the second or third most abundant isomiRs.

**Figure 3:**
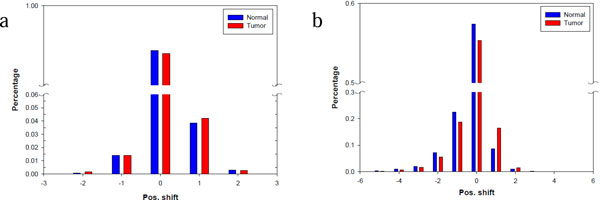
**Position shifts at the 5' end and 3' end of miRNA reads**. The position shifts at the miRNA 5' end and 3' end can be measured by comparing with the location of the miRBase reference miRNAs. (a) The position shift at the 5' end is less diverse and highly dominated by "0"; (b) the position shift at the 3' end is more diverse

Although highly expressed miRNAs tend to have more isomiR types, the isomiR distribution between different libraries can differ [10,13], indicating their diverse regulatory roles. The present study's data showed that position shifts occurred more frequently at the 3' end of miRNAs. Detailed analysis of the position shifts in miRNAs at both ends revealed that the 2 libraries displayed similar patterns in position shift (Figure 3). At the 5' end, position shift "0" dominated, contributing 95% of all miRNA reads; position shift "-1" and "1" accounted for only 1% and 4% of miRNA reads. At the 3' end, position shift "0" contributed 56% of all miRNA reads, remaining dominant. Other position shifts "-2," "-1," "1," and "2" accounted for 5%, 20%, 15%, and 1% of miRNA reads, respectively. In summary, most isomiRs displayed higher frequency position shifts at their 3' end during the miRNA maturation procedure. Additional file 6 displays the overall mapping results of the miRNAs of all libraries.

### Arm selection preferences in breast cancer

**Figure 4:**
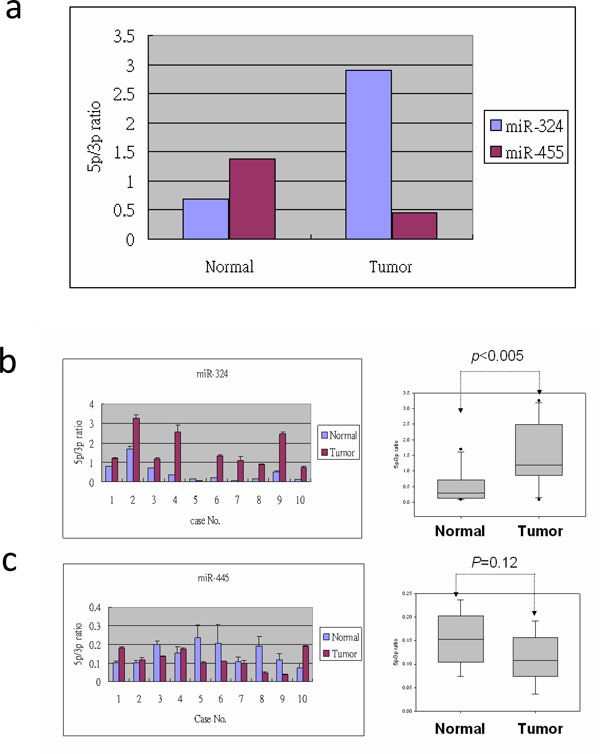
**The 5p/3p arm selection of miR-324 and miR-455 in 10 breast cancer patients**. (A) Ratios of 5p and 3p arms of miR-324 and miR-455 as observed from NGS data. (B) and (C) Ratios of 5p and 3p arms of miR-324 and miR-455 in the breast cancer tissues of 10 patients as evaluated using stem-loop quantitative PCR (left panels). Ratios of 5p and 3p arm usage are indicated in the boxplots in the right panels. All samples were assessed in triplicate and analyzed using Student's t test (p < 0.05 was considered significant)

According to miRBase annotation, several pre-miRNAs encode mature miRNAs at both arms; however, several encode mature miRNAs at only one arm. With increasing NGS sequencing depth, additional miRNAs can be detected at the opposite arms of the pre-miRNAs, originally annotated to encode mature miRNAs at only one arm [10,13]. This phenomenon was observed by mapping the clean reads back to pre-miRNAs (Table 1). Figure 2 presents one case in which hsa-miR-511-1 was annotated to encode mature miRNA at its 5p arm according to miRBase 17. However, additional miRNA was detected at its 3p arm. The newly detected 3p miRNA displayed higher expression levels than the original 5p miRNA. Squadrito et al. [57] reported that miR-511-3p is the major active strand of miR-511 in tumor-associated macrophages. In NGS analysis, results showed a higher abundance of miR-511-3p than miR-511-5p in breast cancer cells. To further investigate regulation of 5p and 3p arm selection of pre-miRNAs during breast cancer progression, the pre-miRNAs annotated to encode mature miRNAs at both arms were selected and their ratios of 5p arm to 3p arm expression were further compared in normal breast and breast tumor tissues. Based on hydrogen bonding theory, the selection preference between the 5p arm and 3p arm of pre-miRNA is an intrinsic characteristic of pre-miRNA. Data indicated that most of the examined pre-miRNAs were compatible with this theory, and the selection preferences of the 5p and 3p arms were consistent in normal and tumor tissues. In 17 of the pre-miRNAs, 5p/3p selection preference was not always consistent during breast cancer progression (Table 4). Stem-loop real-time PCR was used to further examine the expression of the 5p arm and 3p arm miRNAs of miR-324 and miR-455 in 10 paired (tumor and adjacent normal) breast cancer tissue samples. In Figure 4, the NGS data showed that the expression ratios of the 5p arm to 3p arm miRNAs of miR-324 and miR-455 were individually increased and decreased in the tumor, respectively, compared with in normal tissue. The 10 paired tissue samples provided consistent results. The ratio of 5p to 3p expression in miR-324 was increased significantly in breast cancer cells. These results showed that the miRNAs of samples did not display 5p or 3p preference consistently, suggesting the existence of another regulatory mechanism. This issue warrants further investigation.

**Table 4:** Arm selection preference of 5p arm and 3p arm miRNAs in normal breast and breast tumor tissues.

pre-miRNA	Location	N5p	N3p	T5p	T3p
hsa-miR-214	mi:30-51; MA:71-92	88	226	133	171
hsa-miR-576	5p:16-37; 3p:55-76	7	44	19	17
hsa-miR-154	MA:15-36; mi:51-72	21	8	7	14
hsa-miR-193a	5p:21-42; 3p:55-76	1 836	1 127	488	2 384
hsa-miR-296	5p:14-34; 3p:48-69	24	34	8	4
hsa-miR-361	5p:6-27; 3p:45-67	105	215	1 043	310
hsa-miR-324	5p:16-38; 3p:53-72	233	341	317	91
hsa-miR-339	5p:15-37; 3p:50-72	215	217	298	194
hsa-miR-493	mi:16-37; MA:57-78	2	5	22	9
hsa-miR-455	5p:16-37; 3p:54-74	99	72	107	308
hsa-miR-664	mi:11-34; MA:49-71	58	8	21	87
hsa-miR-212	3pM:71-91	22	7	7	6
hsa-miR-142	5p:16-36; 3p:52-74	914	1 102	8 661	10 074
hsa-miR-362	5p:5-28; 3p:42-63	174	34	145	114
hsa-miR-376a-1	mi:7-28; MA:44-64	41	24	9	13
hsa-miR-382	5pM:11-32	217	23	51	30
hsa-miR-151	5p:11-31; 3p:47-67	946	1 249	2 721	3 109

N5p, N3p, T5p, and T3p denote miRNA relative read counts (TPM) of 5p arm and 3p arm in normal and tumor tissues

## Conclusions

The present study performed a series of sequence analysis to evaluate miRNA-associated changes in breast cancer, including miRNA expression, arm selection, 3' end modifications, and position shifts in isomiRs. We identified 22 differentially expressed miRNAs in normal breast and breast tumor tissue that might be involved in breast cancer progression through regulation of MAPK signaling. MicroRNAs widely displayed 3' end modifications and position shifts in isomiRs in breast cancer. However, no significant differences emerged between normal breast and breast tumor tissue during carcinogenesis. Arm usage of some miRNAs displayed significant preferences in breast cancer, suggesting that hydrogen bonding theory does not sufficiently explain 5p or 3p arm selection during carcinogenesis. Further investigation of the possible effects of arm selection of miRNAs on breast carcinogenesis is needed. The present study's findings provide insights into breast cancer that might facilitate the development of future cancer therapy.

## Competing interests

The authors declare that they have no competing interests.

## Authors' contributions

HTC and SCL executed this study and prepared the draft of the manuscript. HWP was responsible for PCR validation of miRNA. MRH performed pathway enrichment analysis. LYH, HWP, and SYY assisted with tissue preparation and RNA extraction. WHL, LPG and KWT supervised the study and edited the manuscript.

## Supplementary Material

Additional file 1 Sequences of primers for miRNA detection Click here for file

Additional file 2 **Expression levels in normal and tumor tissues**. Click here for file

Additional file 3 **The enriched pathway of the target gene union of hsa-miR-141 and hsa-miR-200b (tumor-preferring)**. The target genes of hsa-miR-141 and hsa-miR-200b were significantly enriched in the MAPK pathway (*p *= 2.1E-6). The target genes are labeled in red Click here for file

Additional file 4 **The enriched pathway of the target gene union of hsa-miR-22, hsa-miR-125b, and hsa-miR-99a (normal-preferring)**. The target genes of hsa-miR-22, hsa-miR-125b, and hsa-miR-99a were significantly enriched in the MAPK pathway (*p *= 2.4E-6). The target genes are labeled in red. Click here for file

Additional file 5 **Distribution of 3' end modifications**. Using the alternative mapping procedure, the 3' end modification events were quantified. In this figure, only the modification events more than 1% in all libraries are illustrated Click here for file

Additional file 6 **Mapping information of miRNA reads to pre-miRNAs in all libraries**. Click here for file
