# Supplementary figures and images for: Comprehensive analysis of microRNAs in breast cancer
Source: BMC Genomics. 2012 Dec 7;13(Suppl 7):S18. doi: 10.1186/1471-2164-13-S7-S18 (PMC3521236; doi:10.1186/1471-2164-13-S7-S18)

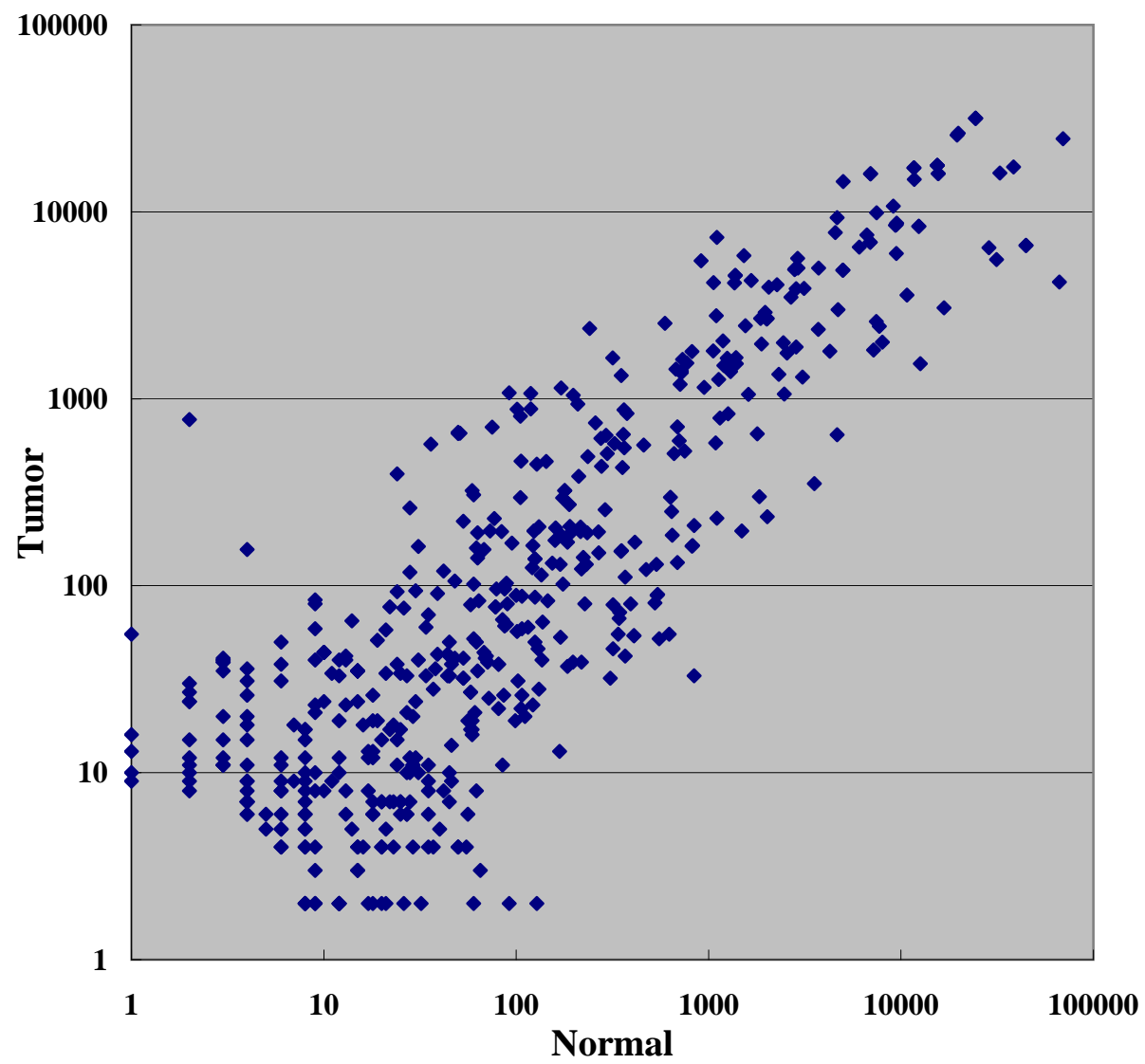

Supplement: Additional file 2 — Expression levels in normal and tumor tissues. [file 1471-2164-13-S7-S18-S2.pdf]

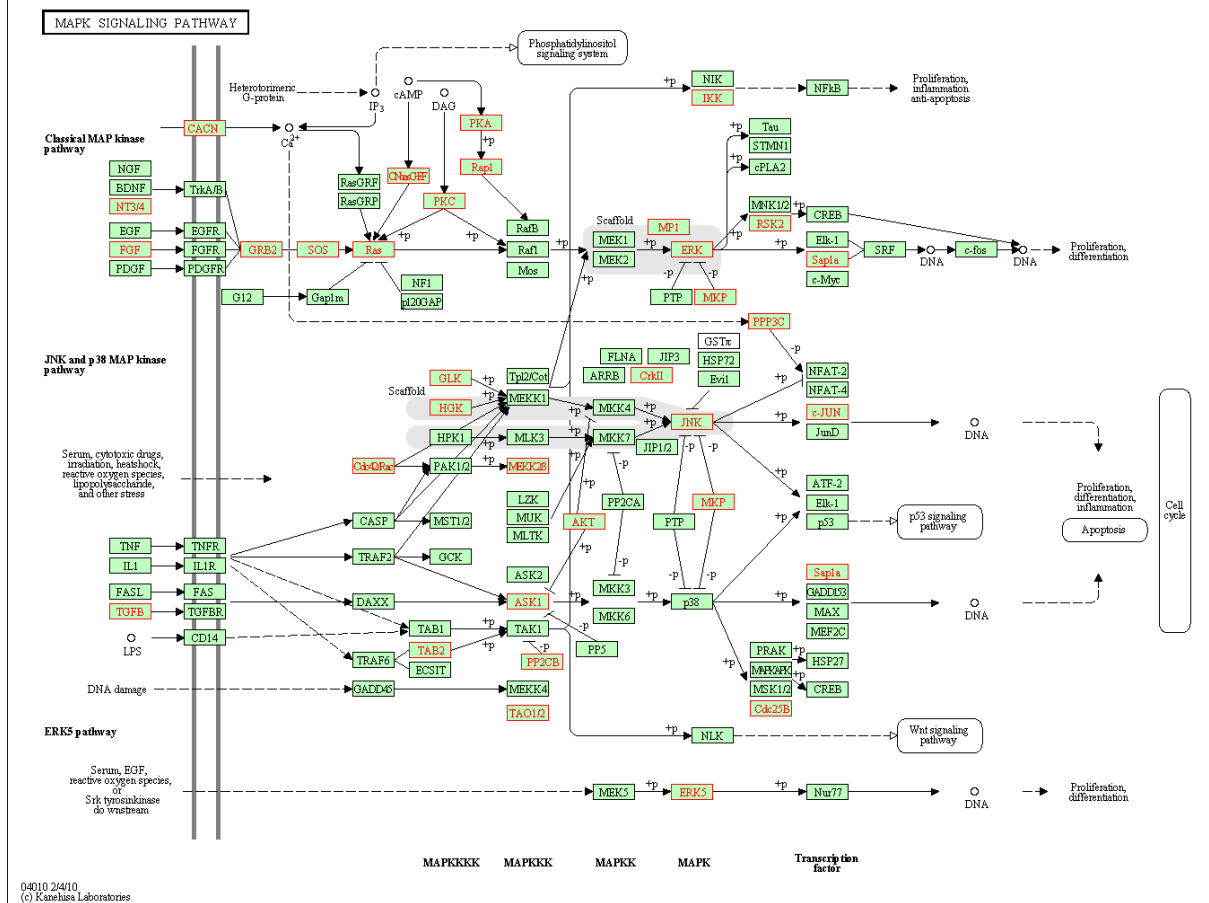

Supplement: Additional file 3 — The enriched pathway of the target gene union of hsa-miR-141 and hsa-miR-200b (tumor-preferring). The target genes of hsa-miR-141 and hsa-miR-200b were significantly enriched in the MAPK pathway (p = 2.1E-6). The target genes are labeled in red [file 1471-2164-13-S7-S18-S3.pdf]

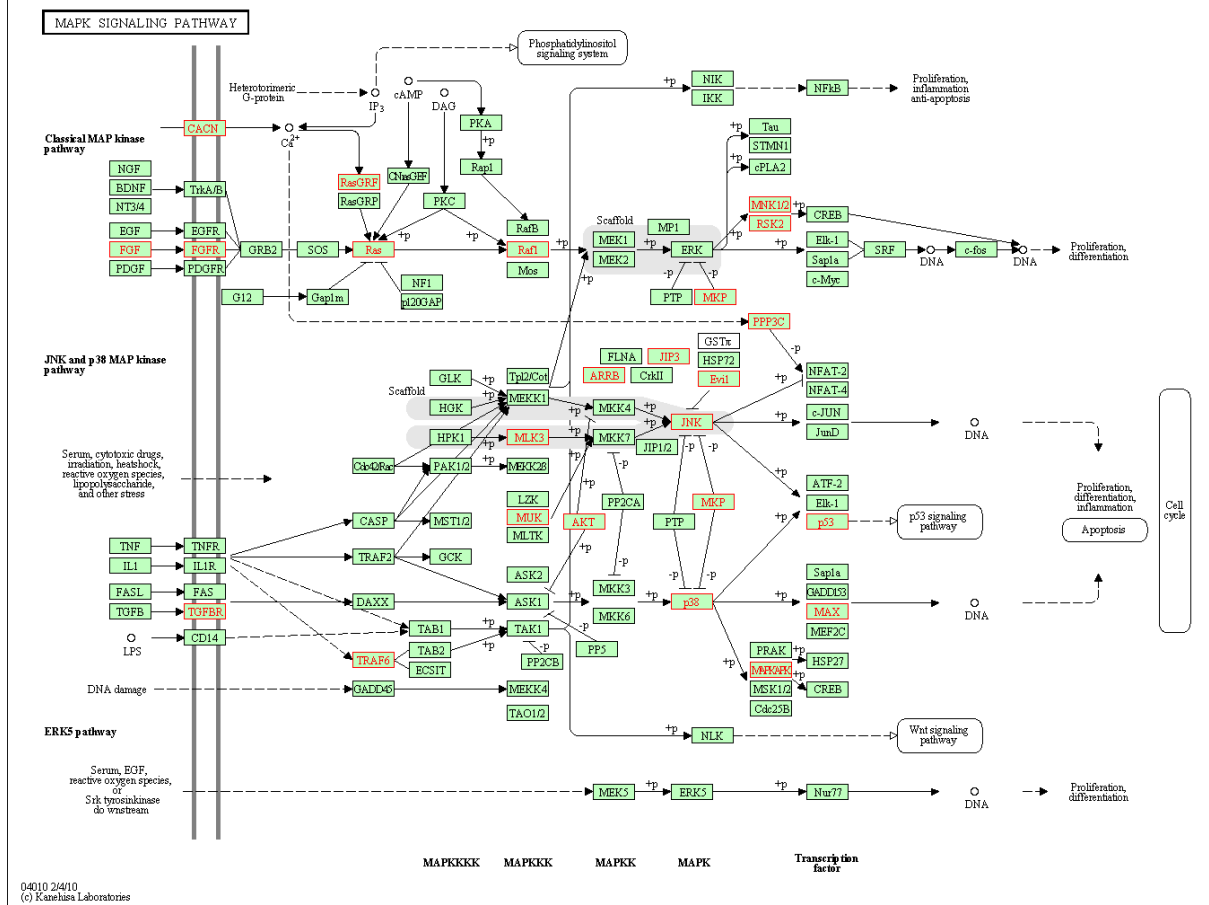

Supplement: Additional file 4 — The enriched pathway of the target gene union of hsa-miR-22, hsa-miR-125b, and hsa-miR-99a (normal-preferring). The target genes of hsa-miR-22, hsa-miR-125b, and hsa-miR-99a were significantly enriched in the MAPK pathway (p = 2.4E-6). The target genes are labeled in red. [file 1471-2164-13-S7-S18-S4.pdf]

**Global modification pattern**

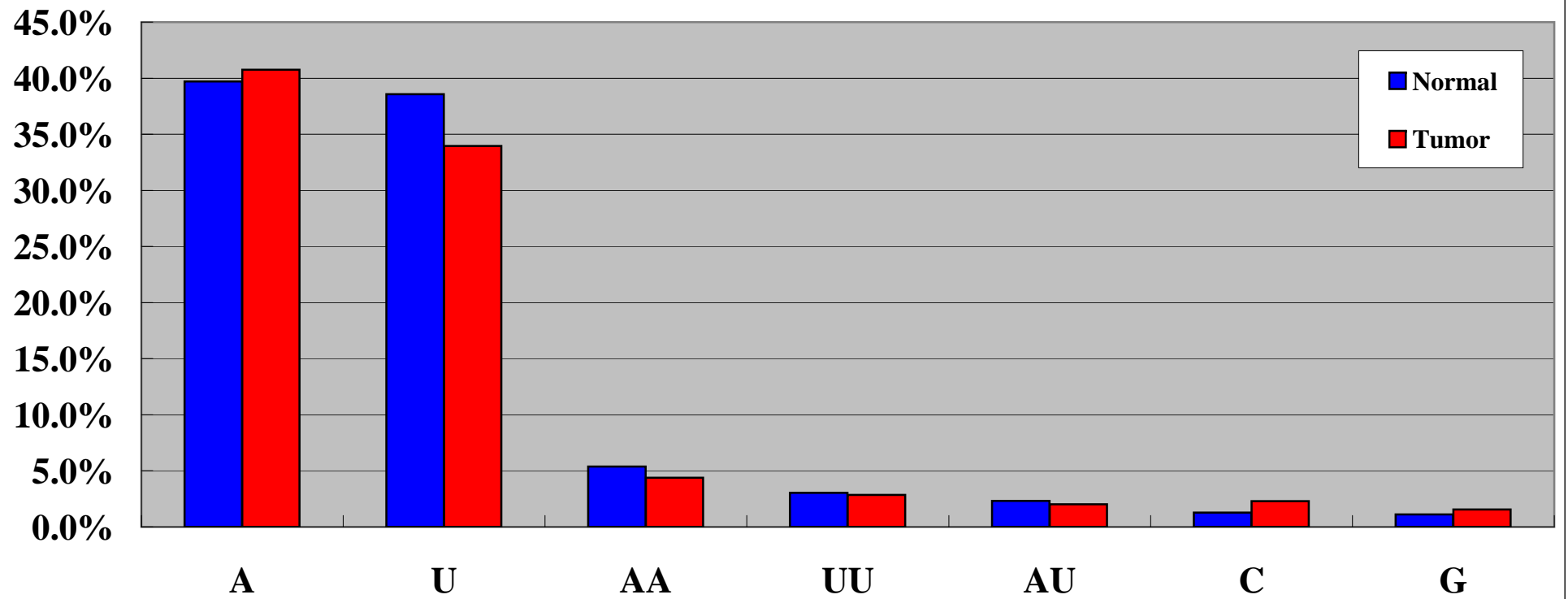

Supplement: Additional file 5 — Distribution of 3' end modifications. Using the alternative mapping procedure, the 3' end modification events were quantified. In this figure, only the modification events more than 1% in all libraries are illustrated [file 1471-2164-13-S7-S18-S5.pdf]
